# Managing Foreign Body Airway Obstruction with Magill Forceps: A Case Report

**DOI:** 10.5811/cpcem.47280

**Published:** 2025-09-10

**Authors:** Ossama Sayed, Samuel Garcia, Benjamin J. Sandefur

**Affiliations:** * Mayo Clinic College of Medicine and Science, Department of Emergency Medicine, Rochester, Minnesota; † Mayo Clinic College of Medicine and Science, Division of Pulmonary and Critical Care Medicine, Rochester, Minnesota

**Keywords:** foreign body airway obstruction, Magill forceps, emergency airway management, video laryngoscopy, case report

## Abstract

**Introduction:**

Foreign body airway obstruction is a high-stakes airway emergency that can rapidly become fatal without timely intervention.

**Case Report:**

We present a case of a 65-year-old male in respiratory extremis due to aspiration of a chicken bone. Following double setup for rapid sequence intubation and cricothyrotomy, the foreign body was successfully removed using Magill forceps under video laryngoscopic guidance.

**Conclusion:**

This case highlights the critical role of early recognition, team readiness, and familiarity with Magill forceps technique in managing foreign body airway obstruction in unstable patients.

## INTRODUCTION

Foreign body (FB) airway obstruction is an airway emergency responsible for 5,200 deaths in the United States annually.^1^ Foreign bodies may lodge in the pharynx, perilaryngeal space, esophagus, larynx, or tracheobronchial tree. Foreign-body impactions are most common in pediatric populations and specific adult groups, including the elderly, incarcerated individuals, and patients with psychiatric disorders, developmental delays, or alcohol intoxication.^2^ In adults, obstruction most commonly occurs from food, often fish or chicken bones.^3^ Complications from FBs in the upper aerodigestive tract are significant. Older patients are at the highest risk, particularly from sharp FBs like fish bones, which necessitates close observation and timely management, especially in the perioperative period.^4^

When caring for patients with a potential airway FB, it is imperative to identify signs and symptoms of partial or complete airway obstruction, such as cough, fever, breathlessness, and wheezing.^5^ These symptoms mimic many other medical conditions; therefore, a high index of suspicion is required when the history is not suggestive.^6^ A frequent symptom in both adults and children is “penetration syndrome,” defined as the sudden onset of choking and intractable cough with or without vomiting.^5^ Given non-specific signs and symptoms, FB airway obstruction may be attributed to other medical diagnoses, especially in the absence of a clear history of aspiration or ingestion. Prompt diagnosis and immediate intervention are required to avoid potentially fatal consequences. We present a case of critical FB airway obstruction that highlights the importance of prompt identification and management. We additionally review management options for upper airway FB removal using Magill forceps.

## CASE REPORT

A 65-year-old male with a medical history of alcohol use disorder presented to the emergency department (ED) following suspected aspiration. He had been eating chicken wings when he appeared to choke and lose consciousness. Emergency medical services found the patient to be altered, stridulous, and in respiratory extremis, with oxygen saturation in the mid-80s. Paramedics made a single, unsuccessful attempt to view the pharynx with a direct laryngoscope without medications. The patient was then transported to the ED with 15 liters per minute (L/min) oxygen delivered via a non-rebreather facemask.

The patient’s vital signs on arrival were heart rate 109 beats per minute, respiratory rate 28 breaths per minute, blood pressure 158/80 millimeters of mercury (mmHg), and oxygen saturation in the low 80s on 15 L/min oxygen. The patient was in a semi-Fowler position, exhibiting high-pitched stridor and markedly increased work of breathing. The patient was altered, with psychomotor agitation with uncoordinated movements, consistent with acute hypoxemia. Bilateral breath sounds were present and symmetric but diminished. Due to concern for FB airway obstruction, we proceeded with a double setup for rapid sequence intubation and cricothyrotomy, readying a C-MAC^TM^ videolaryngoscope (Karl Storz SE & Co. KG, Tuttlingen, Germany), Magill forceps, double suction, and a cricothyrotomy kit.

**Image 1A:**
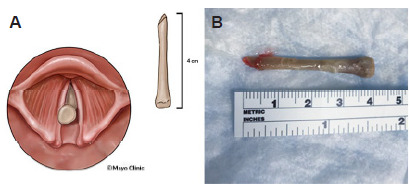
Medical illustration showing the anatomical position of the foreign body lodged within the glottic opening. **Image 1B**. Extracted foreign body measuring approximately 4 cm in length, identified as a partial chicken bone with one sharp end, which was retrieved from the glottic aperture using Magill forceps.

We emergently activated our airway backup protocols, mobilizing anesthesiology, otorhinolaryngology (ENT), and general surgery services. We determined that a “forced-to-act” scenario existed, mandating an initial best attempt at FB removal and intubation under neuromuscular blockade, with a plan for emergency cricothyrotomy should the first attempt fail. We passively preoxygenated the patient with 100% oxygen via an anesthesia bag and mask, achieving a saturation of 99%. We did not administer positive pressure ventilation due to concern that the FB could be pushed deeper into the airway. Using etomidate and succinylcholine in standard doses to achieve rapid sequence intubation conditions, we carefully introduced a Macintosh 4 videolaryngoscope into the mouth. Copious pharyngeal secretions were present, which we suctioned. We observed a white, glistening FB protruding from the glottic aperture. Using Magill forceps, we cautiously approached and grasped the FB, which was successfully removed (Image 1A). We intubated the trachea with a 7.5 endotracheal tube and confirmed placement with colorimetric capnography, bilateral breath sounds, and chest radiography. A chest radiograph revealed no radiopaque FBs. Propofol and fentanyl were used for post-intubation sedation.

The extracted FB measured 4 centimeters (cm) and was sharp on one end, identified as a partial bone from poultry (Image 1B). We administered empiric ceftriaxone, metronidazole, and dexamethasone, given concern for posterior trachea penetrating injury, and we admitted the patient to the medical intensive care unit, where he remained intubated overnight. On hospital day 2, the patient was extubated. The ENT subsequently performed a comprehensive upper airway exam using a flexible fiberoptic scope under topical anesthesia. They noted a hyperemic area on the posterior left vocal cord with a small amount of edema. The glottis and trachea were otherwise unremarkable. The patient was discharged home in stable condition with a five-day course of corticosteroids and a seven-day course of prophylactic amoxicillin-clavulanate.

*CPC-EM Capsule*

What do we already know about this clinical entity?

*Foreign body airway obstruction is a high-stakes emergency requiring rapid diagnosis and intervention to prevent fatal hypoxia*.

What makes this presentation of disease reportable?

*This case highlights successful Magill forceps extraction of a chicken bone in an unstable patient with impending complete obstruction*.

What is the major learning point?

*Magill forceps can be lifesaving in upper airway obstruction; proficiency requires both familiarity with the tool and practiced versatility in grip technique*.

How might this improve emergency medicine practice?

*Early recognition, team readiness, and Magill forceps proficiency are vital skills in emergent airway management*.

## DISCUSSION

**Image 2:**
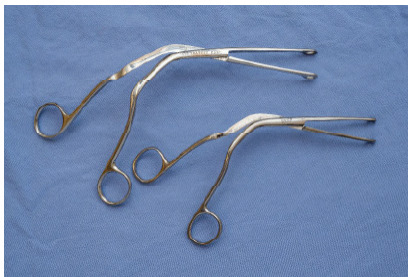
The Magill forceps is a stainless-steel instrument used in airway procedures, characterized by an angled design and serrated jaws.

The management of patients with partially or completely obstructing airway FBs can present a challenge for emergency physicians. Incomplete FB airway obstruction can rapidly degenerate into complete obstruction either spontaneously or inadvertently from airway management techniques, such as mask ventilation. While incomplete FB airway obstruction in stable patients is best managed with expedited specialty care in an operating room (OR) setting, incomplete obstruction in unstable patients (eg, severe hypoxemia, agitation, rapid deterioration) must be rapidly addressed in the ED. In the described case, an incomplete FB airway obstruction from a poultry bone was successfully relieved from a patient in extremis using Magill forceps. The Magill forceps is a stainless-steel surgical instrument with a long, angulated shaft and blunt, serrated jaws (Image 2).

The handle is designed to close the jaws when closing the handle, with the articulation midway along the length of the tool. Magill forceps are used to manipulate tracheal tubes, gastric tubes, and throat packs, although in the emergency setting, their primary utility is retrieval of upper airway FBs. The Magill forceps technique can be used for extraction of different types of FBs within reach of the forceps and in any age group. A 2020 systematic review by Couper et al identified Magill forceps as one of the three key interventions associated with FB airway obstruction survival with good neurological outcome.^7^ To clear a FB airway obstruction located in the oropharynx or hypopharynx involves several critical steps to ensure the safe and effective removal of the obstructing object. While we focus on the management of FB airway obstruction with Magill forceps, it is important to note that a well-considered and stepwise management approach to this type of obstruction is crucial but not covered in detail here. Detailed discussions of this topic for adult and pediatric patients can be found in dedicated emergency airway management texts.

### Patient Populations

Two broad patient populations present with FB airway obstructions: those requiring immediate intervention (eg, complete obstruction or incomplete obstruction in extremis); and those who are stable and candidates for awake intervention. For those in extremis, a “forced-to-act” scenario often exists, and the clinician must determine whether a single best attempt with sedation and neuromuscular blockade is indicated, with a plan for immediate cricothyrotomy should the attempt fail. In the stable population, strong consideration should be given for a direct-to-OR approach. An awake procedure should be considered in patients who can maintain their own airway patency, spontaneously ventilate, follow commands, and who require no more than anxiolysis to cooperate.^8^ Intravenous access should be established. All patients should be placed on cardiac monitor and pulse oximetry and properly preoxygenated, avoiding positive pressure ventilation when possible, which may lead to abrupt complete FB airway obstruction. Below we outline and contrast the steps in managing FB airway obstruction with Magill forceps in a patient in extremis vs in the stable patient.

### Preparation, Setup, and Technique

**Image 3A:**
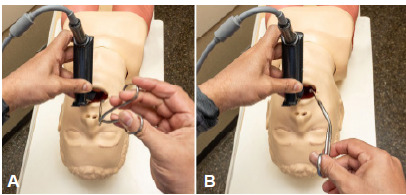
Demonstration of the “overhand method” of holding Magill forceps, with the handle superior and jaws directed downward for ergonomic access to the oropharynx. **Image 3B**. Demonstration of the “traditional method” of holding Magill forceps, commonly used in posterior oropharyngeal procedures such as tracheal tube or nasogastric tube manipulation.

1. Preparing the Patient: We recommend meticulous passive preoxygenation, avoiding positive pressure ventilation, which could worsen the obstruction. For those in extremis, place the patient in a sniffing position. For an awake approach, the patient should be positioned as best tolerated; cooperation is vital to successful outcomes.
2. Sedating and Anesthetizing the Patient: For those who cannot be temporized and require immediate intervention due to suspected near complete obstruction or complete obstruction, a “forced-to-act” scenario exists. In this extreme circumstance, we recommend proceeding with induction and complete neuromuscular blockade, allowing for a “best attempt” with a staged approach including FB extraction with Magill forceps or suction, pushing the FB distally into a mainstem bronchus, or cricothyrotomy, as the circumstances dictate. This contrasts with the awake patient who is actively maintaining airway patency, ideally cooperative, in whom a direct-to-OR plan or ED awake procedure should be considered. In this circumstance, we administer 0.3 mg of IV glycopyrrolate to reduce oral secretions and enhance topical anesthesia effectiveness, following which we atomize 4% lidocaine as a topical anesthetic.^8^ After appropriate topical analgesia, many patients may require little to no sedation to proceed with careful awake laryngoscopy. Sedative medication, if required, should be administered with a goal of decreasing periprocedural anxiety rather than sedation. We recommend using 1 mg boluses of midazolam for this purpose.^8^
3. Visualizing the Airway: We use a standard geometry videolaryngoscope to carefully visualize the airway in both subsets of patients. When feasible, identification of the FB with endoscopic visualization (ie, nasopharyngoscopy) is useful for planning. While a hyperangulated geometry videolaryngoscope may allow for an easier view of the glottis or FB in either subset of patients, we favor standard geometry blades as it most closely approximates Magill forceps geometry. In all circumstances, measured and incremental movements toward the glottic structures are advised, to prevent inadvertent dislodgment, which could result in complete obstruction.
4. Holding the Magill Forceps: Two techniques for holding Magill forceps during FB extraction are demonstrated in Images 3A and 3B. In the overhand method (Image 3A), the forceps are held with the right thumb and middle finger, positioning the hand superior to the instrument in a handshake grip, with the angle of the forceps directed downward. This approach can help keep the operator’s hand out of the line of sight and may align more closely with the oral and pharyngeal axes. The traditional method (Image 3B), often used in posterior oropharyngeal procedures—such as tracheal tube or nasogastric tube advancement—positions the forceps at a different angle relative to the hand and is suited for varied procedural contexts. Both techniques may offer distinct ergonomic and visual advantages depending on the clinical scenario and the operator. Despite their critical utility, standardized education on Magill forceps technique is lacking, with recent work highlighting wide variability in hand positioning and significant differences in FB removal times depending on the grip used.^9^
5. Removing the Foreign Body with Magill Forceps: After incremental insertion of the laryngoscope, the FB is visualized, carefully grasped using Magill forceps, and then removed. Suction may be required to clear airway secretions for better visualization. For patients in extremis, secure the airway by passing the appropriately sized endotracheal tube into the trachea and confirm placement by colorimetric capnography, bilateral breath sounds, and chest radiography. Chest radiography is also an important modality for excluding additional aspirated FBs in some circumstances. For awake patients, securing the airway may not be necessary. In this circumstance, after the FB is removed, we recommend consideration of nasopharyngoscopy to ensure no residual FBs remain and to assess for injury.^10^

## CONCLUSION

Foreign body airway obstruction is a potentially fatal airway emergency that demands rapid identification and management. In such scenarios, Magill forceps can be a lifesaving tool when used by skilled operators following a systematic approach. Although the choice of holding technique is less critical than tool familiarity and a structured approach, further study is needed to clarify best practice. Hands-on training to develop proficiency, such as on manikin or cadaveric models, will ensure the clinician can act confidently in emergencies, minimize risks, and improve patient outcomes.
